# Neighbourhood child population density as a proxy measure for exposure to respiratory infections in the first year of life: A validation study

**DOI:** 10.1371/journal.pone.0203743

**Published:** 2018-09-12

**Authors:** Judith E. Lupatsch, Christian Kreis, Insa Korten, Philipp Latzin, Urs Frey, Claudia E. Kuehni, Ben D. Spycher

**Affiliations:** 1 Institute of Social and Preventive Medicine, University of Bern, Bern, Switzerland; 2 Institute of Pharmaceutical Medicine, University of Basel, Basel Switzerland; 3 Division of Respiratory Medicine, Department of Pediatrics, Inselspital, Bern University Hospital, University of Bern, Bern, Switzerland; 4 University of Basel, Children’s Hospital (UKBB), Basel, Switzerland; Kliniken der Stadt Köln gGmbH, GERMANY

## Abstract

**Background:**

Assessing exposure to infections in early childhood is of interest in many epidemiological investigations. Because exposure to infections is difficult to measure directly, epidemiological studies have used surrogate measures available from routine data such as birth order and population density. However, the association between population density and exposure to infections is unclear. We assessed whether neighbourhood child population density is associated with respiratory infections in infants.

**Methods:**

With the Basel-Bern lung infant development study (BILD), a prospective Swiss cohort study of healthy neonates, respiratory symptoms and infections were assessed by weekly telephone interviews with the mother throughout the first year of life. Using population census data, we calculated neighbourhood child density as the number of children < 16 years of age living within a 250 m radius around the residence of each child. We used negative binomial regression models to assess associations between neighbourhood child density and the number of weeks with respiratory infections and adjusted for potential confounders including the number of older siblings, day-care attendance and duration of breastfeeding. We investigated possible interactions between neighbourhood child population density and older siblings assuming that older siblings mix with other children in the neighbourhood.

**Results:**

The analyses included 487 infants. We found no evidence of an association between quintiles of neighbourhood child density and number of respiratory symptoms (p = 0.59, incidence rate ratios comparing highest to lowest quintile: 1.15, 95%-confidence interval: 0.90–1.47). There was no evidence of interaction with older siblings (p = 0.44). Results were similar in crude and in fully adjusted models.

**Conclusions:**

Our study suggests that in Switzerland neighbourhood child density is a poor proxy for exposure to infections in infancy.

## Introduction

Measuring exposure to infections in early childhood is of interest in many epidemiological investigations. The ‘hygiene hypothesis’ postulates that a low infectious burden in early childhood is associated with an increased risk of allergic diseases such as asthma or allergic rhinitis and of autoimmune diseases such as type 1 diabetes[1–3]. Frequent infection with specific viruses might also act as a driver of asthma development [4]. The timing of exposure to early childhood infections is also discussed as an important factor in the aetiology of childhood leukaemia [5].

Because exposure to infections is difficult to measure directly, epidemiological studies typically rely on surrogate measures such as day-care attendance, number of older siblings and, less commonly, population density. These measures differ in their validity and in their ease of assessment. While it is known that children attending day-care centres [6,7] or children with older siblings [8] tend to have more infections, evidence of associations between population density and children’s exposure to infections is limited. To our knowledge, only one previous ecologic study from the UK has investigated this and found an increase in hospital admissions for infection in children living in areas with high population density [9]. While day-care attendance can only be assessed through active participation of parents (interview or questionnaires), population density and birth order are more readily available from routine data. The latter are thus not only easier to assess but also allow avoiding the risk selection and recall bias. While higher birth order is a valid surrogate for infectious exposure, to our knowledge, no previous study has investigated if the same is true for higher population density.

The number of other children in the neighbourhood is a plausible surrogate of a child’s exposure to infections for several reasons. First, children’s exposure to infections is mostly through contact with other children. Studies using social contact matrixes could demonstrate that schoolchildren have a particularly high frequency of contacts, especially with other schoolchildren [10–12]. However, frequent close contacts between children of similar ages are also likely to occur among children from the same neighbourhood. Second and related to this, a higher child population density might lead to a higher circulation of infections in a neighbourhood. Mathematical models based on such contact data suggest that schoolchildren drive the initial phase of an epidemic of infections transmitted by droplets or through close contacts [10]. Frequent neighbourhood contacts between schoolchildren could thus lead to a rapid spread of epidemics through neighbourhoods with high child density. Infections circulating in a neighbourhood can be transmitted to infants through close contacts with older siblings.

We aimed to determine whether infants living in neighbourhoods with high child population density have more frequent respiratory infections than other children using data from the Basel-Bern Infant Lung Development (BILD) cohort study. In this ongoing birth cohort study, respiratory symptoms and infections are assessed prospectively throughout the first year of life through weekly telephone interviews with parents [13]. We also assessed whether the hypothesised association between child population density and respiratory infections is modified by having older siblings.

## Materials and methods

### Population

The BILD Cohort [13–16] (www.bild-cohort.ch) is an ongoing prospective birth cohort study including unselected infants born after April 1999 in the regions of Bern and Basel, Switzerland. Details of the study design are provided elsewhere [13]. In short, pregnant women are recruited in maternity hospitals and practices of obstetricians. A first interview in the first month after birth included questions on socio-demographic factors as well as prenatal and postnatal exposure to putative risk factors for respiratory disease. During the first year of life of the index child, detailed information on respiratory symptoms was collected through weekly telephone interviews. These interviews also addressed breastfeeding and day care attendance. In the current analysis we included all children in the BILD study from the region of Bern with available geocoded addresses at birth and, as of August 2015, complete information on their first year of life (at least 49 weeks of follow-up).

### Outcomes

The information obtained through weekly interviews included standardised day-time and night-time symptom scores for lower respiratory symptoms (any of the following: cough, wheeze or breathing difficulties). Possible answers were: (0) none, (1) slight; no treatment given (day) or sleep not disturbed (night), (2) required treatment but no outside help (day) or sleep disturbed once but no help required (night), (3) severe; required help (GP) (day) or sleep disturbed more than once or child needed help (night), (4) very severe; admitted to hospital (day) or sleep very disturbed or GP called (night). We calculated the following outcomes from these scores:

- Number of weeks with any respiratory symptoms (day-time or night-time symptom score ≥1).
- Number of weeks with severe respiratory symptoms (day-time or night-time symptom score ≥3).

Lower respiratory tract infections were defined as: cough, wheeze and/or breathing difficulties in combination with upper respiratory tract symptoms or elevated temperature for more than two consecutive days. We calculated the following outcomes:

- Number of weeks with lower respiratory tract infection.
- Number of weeks with lower respiratory tract infection with fever.

### Primary exposure: Child population density

Our main exposure of interest was neighbourhood child population density calculated as the total number of children aged 0–15 years residing within 250 m from the infant’s home at birth. We chose 250 m a priori assuming that this would reflect a typical perimeter within which neighbourhood friends (e.g. of an infant’s older siblings) would live. We obtained geocoded locations of residence at the time of national censuses in 1990, 2000 and 2010 for the entire Swiss resident population from the Swiss National Cohort study [17]. For infants born in a census year, neighbourhood child population density was calculated based on that census. For infants born between two census years, a linear interpolation of the values calculated in these censuses was used, while for children born after 2010 exposure was calculated based on the 2010 census.

### Competing exposures and potential confounders

As competing exposures (presumed to be associated with infections but not or only weakly associated with the main exposure) we also considered the number of older siblings (0, 1, 2+), any day-care attendance during the first year of life (yes / no) and breastfeeding (never, ≤ 6 month, > 6 month). We considered potential confounding of the association between our primary exposure and outcomes by degree of urbanity (urban, rural) and neighbourhood socioeconomic position (quintiles of the Swiss-SEP) [18]. In order to isolate a potential effect of population density on respiratory symptoms mediated through air pollution, we also adjusted our analyses for modelled ambient NO_2_ concentrations (quintiles) at the infants’ residence at birth [19,20].

### Statistical analysis

We assessed incidence rate ratios (IRR) and 95% confidence intervals (CI) for the outcomes comparing quintiles of neighbourhood child density (lowest quintile as reference) using negative binomial regression to account for overdispersion in the number of reported infectious symptoms. We fitted univariable (crude) models separately for potential determinants of early infections (exposures, potential confounders and NO_2_) and multivariable (adjusted) models containing all potential determinants of infections simultaneously. In a second step, we fitted models with an interaction term between child population density (below and above median) and number of siblings. All models were fitted using STATA 13.1 (StataCorp, Texas).

In sensitivity analyses we fitted crude and adjusted models using alternative definitions of neighbourhood population density: First, we set the radius used to define an infant’s neighbourhood to 100 m and 500 m, respectively. Second, we decreased and increased the upper age limit to include only young children below 10 years of age and all residents living in an infant’s neighbourhood, respectively.

## Results

### Population

**Fig 1:**
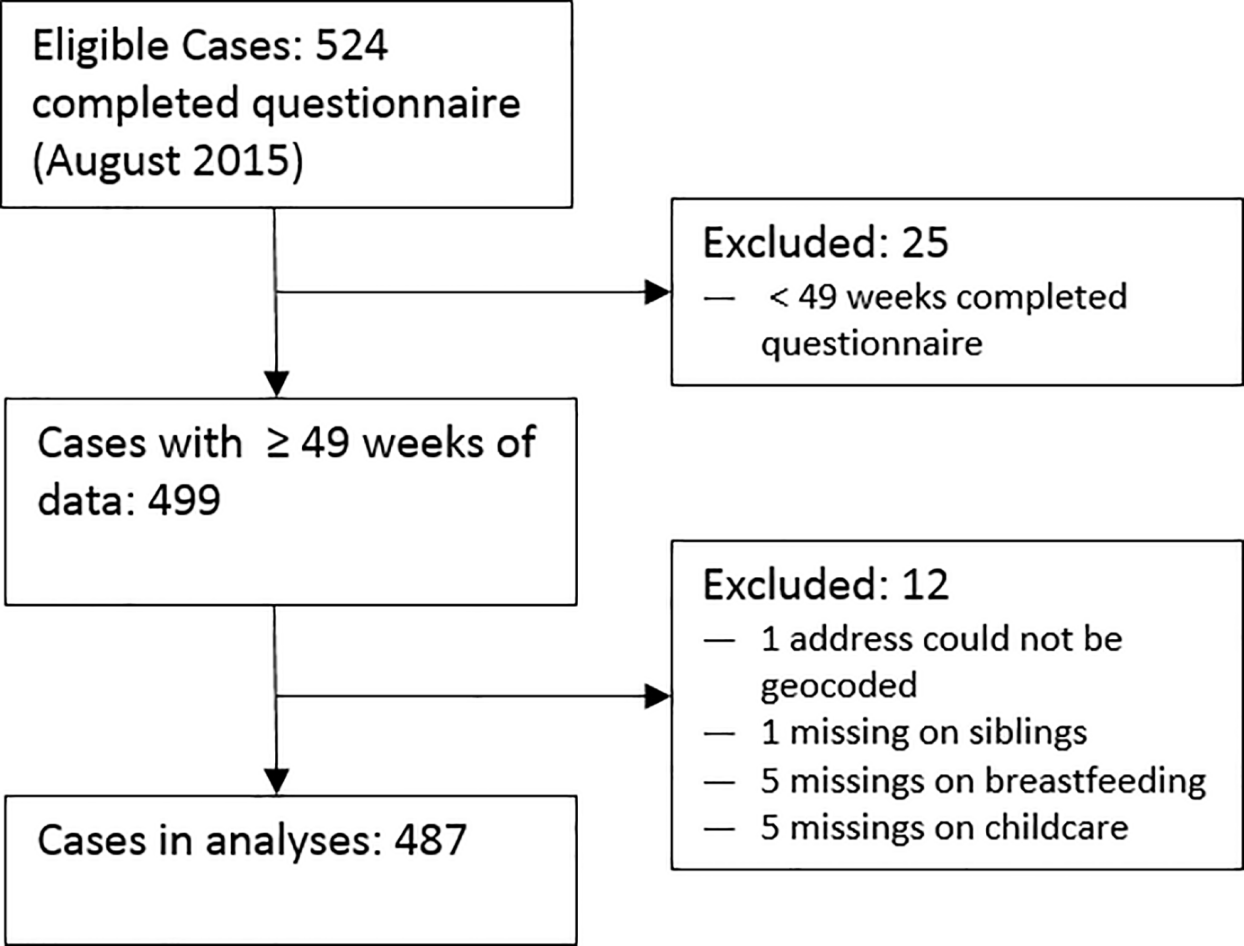
Flow chart of study population.

As of August 2015, weekly interviews during the first year of life were completed for 524 children of the BILD study. After excluding those with less than 49 weeks of follow-up and those with missing data on exposures, we included 487 infants in the analysis (Fig 1)

### Characteristics of the study population

**Fig 2:**
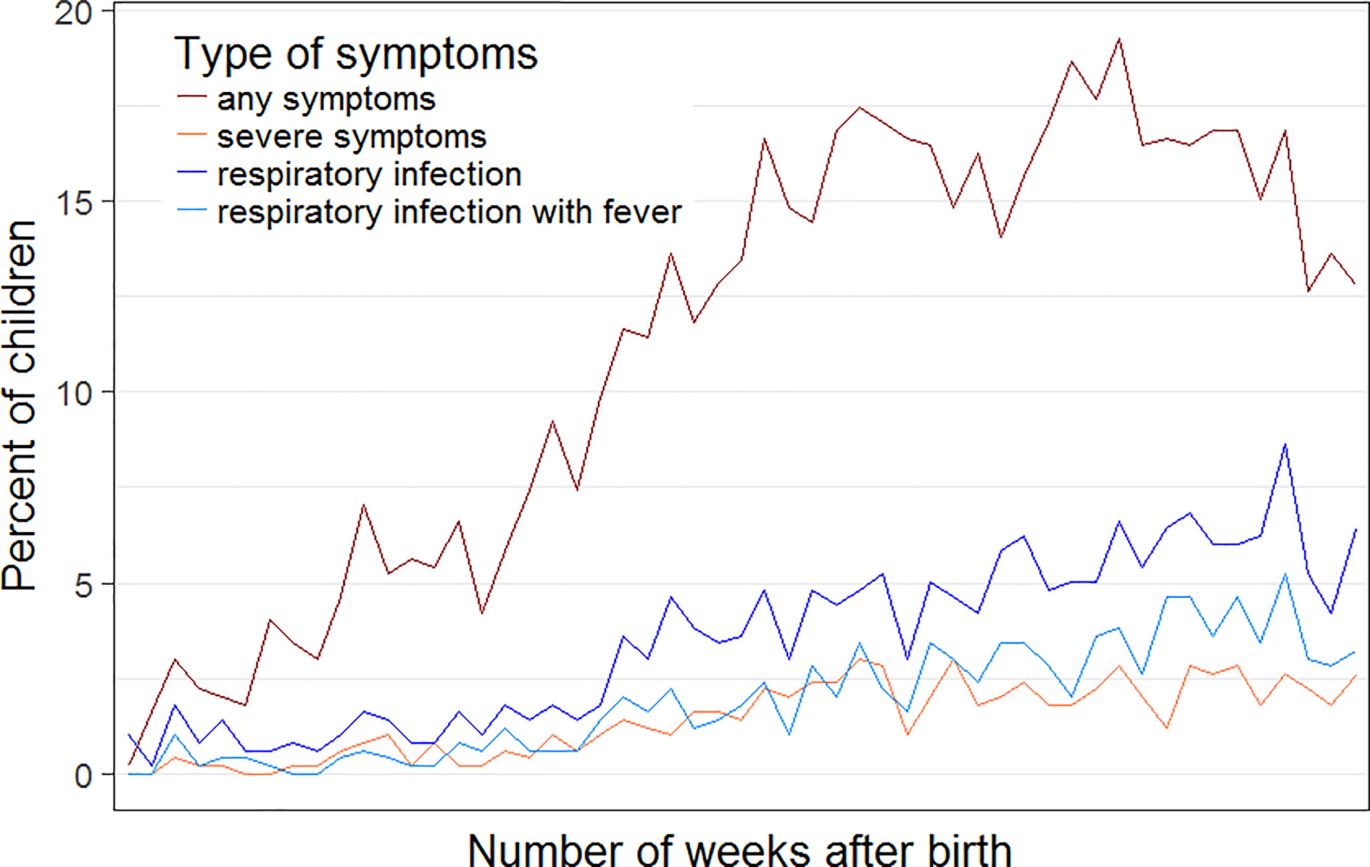
Percentage of children having respiratory symptoms by week of life.

The sample included more girls (54%) than boys (46%) (Table 1). Birthweight (median: 3390 gram) and gestational age (median: 39.9) were within the normal ranges. About one fourth of the children attended day-care during the first year of life (23.5%). The vast majority (98%) of children were breastfed and most (69%) were breastfed for more than 6 months. Most children of this sample lived in urban areas (77.3%). Median ambient annual average NO_2_ concentration at place and time of birth of 20 μg/m^3^ (the annual limit value is set at 30 μg/m^3^ by the Swiss Federal Office for the Environmental [19] (Table 1). Median neighbourhood child density was 50 children within a 250 meter radius (range 0–199). Children living in areas with high child density (above median) were more likely to attend day-care, live in urban areas or in neighbourhoods with high SEP, and to be exposed to high levels of NO_2_ (Table 1). The median number of weeks with any respiratory symptoms was 4 (range 0–24). The frequency of lower respiratory tract infections increased with age (Fig 2). The percentage of children with any respiratory symptoms in a given week after birth rose to above 15% by the 28^th^ week of life and levelled off thereafter (Fig 2).

**Table 1:** Characteristics of included infants from the BILD cohort.

Characteristics		Total		Low child density^a^		High child density^a^		p^e^
**Sex**								
male	n (%)	226	(46.4)	114	(46.9)	112	(45.9)	0.823
female	n (%)	261	(53.6)	129	(53.1)	132	(54.1)	
**Year of Birth**								
1999–2004	n (%)	183	(37.6)	89	(36.6)	94	(38.5)	0.005
2005–2009	n (%)	162	(33.3)	96	(39.5)	66	(27.0)	
2009–2014	n (%)	142	(29.2)	58	(23.9)	84	(34.4)	
**Birth weight (grams)**	median (range)	3390	(1800–4915)	3400	(1960–4915)	3375	(1800–4600)	0.497
**Gestational age (weeks)**	median (range)	39.9	(32–42)	39.9	(32–42)	39.9	(33–42)	0.617
**Child density**^**a**^	median (range)	48.8	(0–199)	24	(0–49)	75.85	(49–199)	NA
**No. Siblings**								
0	n (%)	214	(43.9)	102	(42.0)	112	(45.9)	0.162
1	n (%)	185	(38.0)	89	(36.6)	96	(39.3)	
2+	n (%)	88	(18.1)	52	(21.4)	36	(14.8)	
**Day-care attendance**^**b**^								
no	n (%)	371	(76.2)	212	(87.2)	159	(65.2)	<0.001
yes	n (%)	116	(23.8)	31	(12.8)	85	(34.8)	
**Breastfeeding**								
Never	n (%)	10	(2.1)	5	(2.1)	5	(2.0)	0.287
< = 6 mths	n (%)	140	(28.7)	62	(25.5)	78	(32.0)	
>6 mths	n (%)	337	(69.2)	176	(72.4)	161	(66.0)	
**SES in quintiles**^**c**^								
1	n (%)	61	(12.5)	41	(16.9)	20	(8.2)	<0.001
2	n (%)	91	(18.7)	58	(23.9)	33	(13.5)	
3	n (%)	81	(16.6)	48	(19.8)	33	(13.5)	
4	n (%)	111	(22.8)	48	(19.8)	63	(25.8)	
5	n (%)	143	(29.4)	48	(19.8)	95	(38.9)	
**Urbanity**								
rural	n (%)	110	(22.6)	101	(41.6)	9	(3.7)	<0.001
urban	n (%)	377	(77.4)	142	(58.4)	235	(96.3)	
**NO2 level**^**d**^								
μg/m^3^	median (range)	20	(7–39)	16.4	(7–39)	23.6	(13–38)	<0.001
								
**Any respiratory symptoms**	median (range)	4	(0–24)	4	(0–24)	5	(0–22)	0.389
**Severe respiratory symptoms**	median (range)	0	(0–11)	0	(0–11)	0	(0–7)	0.493
**Lower respiratory tract infection**	median (range)	1	(0–12)	1	(0–12)	1	(0–10)	0.255
**Lower respiratory tract infection with fever**	median (range)	1	(0–11)	1	(0–11)	1	(0–5)	0.978

^a^ number of children within a 250m radius around the residence of the child; High/low refer to above and below median child density

^b^ any day-care attendance during first year of life

^c^ area based socio-economic position of the household

^d^ modelled annual average NO_2_ concentrations measured at place of birth (in μg/m^3^)

^e^ χ^2^ test for categorical variables and rank sum test for continuous variables

### Association between neighbourhood child population density and respiratory infections

There was little evidence of association between neighbourhood child population density and any respiratory symptoms (p likelihood ratio (LR) test: 0.59) (upper part of Table 2). Although incidence tended to be higher in the 2^nd^-5^th^ quintile of child population density than in the first, there was no indication of a dose-response (IRR 2^nd^ quintile: 1.19, 95%CI: 0.93–1.52; IRR 5^th^ quintile: 1.15, 95%CI: 0.90–1.47). Results were similar in the adjusted model (p LR test: 0.57). The IRRs for lower respiratory tract infections were not suggestive of an increased risk with higher neighbourhood child population density neither in the crude nor in the adjusted model (lower part of Table 2). There was a weak association between neighbourhood child density and severe respiratory symptoms which was due to an increased risk in the 2^nd^ quintile (IRR crude 2^nd^ quintile: 1.65, 95% CI: 1.06–2.55) (online supplement S1 Table). No evidence of an association was found for lower respiratory tract infections with fever (S1 Table).

**Table 2:** Risk factors for respiratory symptoms.

		Number of infections		Crude models			Adjusted models^a^		
Risk factor		Median	Range	IRR^b^	95%CI^c^	p^d^	IRR^b^	95%CI^c^	p^d^
**Any respiratory symptoms**		** **	** **	** **	** **	** **	** **	** **	** **
Neighbourhood	1	4	(0–23)	1.00		0.594	1.00		0.571
child population	2	4	(0–24)	1.19	(0.93, 1.52)		1.23	(0.93, 1.62)	
density (250m)	3	4	(0–22)	1.08	(0.85, 1.39)		1.06	(0.78, 1.44)	
in quintiles^e^	4	5	(0–17)	1.19	(0.93, 1.52)		1.11	(0.81, 1.53)	
	5	4	(0–22)	1.15	(0.90, 1.47)		1.17	(0.84, 1.64)	
No. Siblings	0	3	(0–22)	1.00		<0.001	1.00		<0.001
	1	6	(0–23)	1.56	(1.32, 1.84)		1.60	(1.35, 1.88)	
	2	6	(0–24)	1.56	(1.27, 1.91)		1.73	(1.40, 2.13)	
Day-care attendance^f^	no	4	(0–24)	1.00		<0.001		1.00	<0.001
	yes	7	(0–22)	1.46	(1.23, 1.74)		1.58	(1.32, 1.89)	
Breastfeeding	never	2.5	(1–10)	0.69	(0.38, 1.23)	0.455	0.59	(0.33, 1.03)	0.179
	< = 6 mths	5	(0–23)	1.00			1.00	
	>6 mths	4	(0–24)	0.96	(0.81, 1.14)		1.00	(0.85, 1.18)	
Urbanity	rural	4	(0–24)	1.00		0.752	1.00		0.213
	urban	5	(0–22)	0.97	(0.81, 1.17)		0.85	(0.66, 1.10)	
SES in quintiles^g^	1	4	(0–24)	1.00		0.769	1.00		0.823
	2	4	(0–22)	1.01	(0.76, 1.35)		0.97	(0.74, 1.28)	
	3	4	(0–17)	0.98	(0.73, 1.31)		0.90	(0.67, 1.21)	
	4	4	(0–22)	0.99	(0.75, 1.30)		0.91	(0.68, 1.21)	
	5	6	(0–21)	1.11	(0.86, 1.45)		1.01	(0.76, 1.35)	
NO_2_ level	1	4	(0–24)	1.00		0.721	1.00		0.205
at place of birth	2	4.5	(0–22)	1.17	(0.92, 1.49)		1.32	(0.98, 1.77)	
in quintiles^h^	3	5	(0–22)	1.13	(0.89, 1.45)		1.29	(0.93, 1.80)	
	4	6	(0–19)	1.12	(0.88, 1.43)		1.20	(0.84, 1.72)	
	5	4	(0–22)	1.05	(0.82, 1.33)		1.08	(0.74, 1.56)	
**Lower respiratory tract infection**									
Neighbourhood	1	1	(0–11)	1.00		0.828	1.00		0.535
child population	2	1	(0–11)	1.14	(0.83, 1.56)		1.08	(0.75, 1.56)	
density (250m)	3	1	(0–12)	1.00	(0.72, 1.38)		0.90	(0.60, 1.35)	
in quintiles^e^	4	2	(0–10)	1.11	(0.81, 1.53)		0.84	(0.55, 1.28)	
	5	1	(0–10)	0.98	(0.71, 1.35)		0.81	(0.52, 1.27)	
No. Siblings	0	1	(0–10)	1.00		<0.001	1.00		<0.001
	1	2	(0–12)	1.54	(1.24, 1.93)		1.62	(1.29, 2.04)	
	2	2	(0–10)	1.67	(1.27, 2.19)		1.91	(1.44, 2.52)	
Day-care attendance^f^	no	1	(0–12)	1.00		<0.001	1.00		<0.001
	yes	2	(0–11)	1.43	(1.14, 1.80)		1.56	(1.23, 1.98)	
Breastfeeding	never	1.5	(0–5)	0.88	(0.41, 1.87)	0.890	0.86	(0.41, 1.80)	0.920
	< = 6 mths	1	(0–11)	1.00			1.00		
	>6 mths	1	(0–12)	0.95	(0.76, 1.19)		1.00	(0.80, 1.24)	
Urbanity	rural	1	(0–11)	1.00		0.548	1.00		0.053
	urban	1	(0–12)	0.93	(0.73, 1.18)		0.71	(0.50, 1.00)	
SES in quintiles^g^	1	1	(0–9)	1.00		0.185	1.00		0.555
	2	1	(0–11)	1.04	(0.72, 1.50)		1.02	(0.71, 1.48)	
	3	1	(0–10)	0.80	(0.54, 1.17)		0.80	(0.54, 1.18)	
	4	1	(0–12)	0.94	(0.65, 1.34)		0.91	(0.62, 1.34)	
	5	2	(0–11)	1.17	(0.83, 1.64)		1.03	(0.70, 1.51)	
NO_2_ level	1	1	(0–10)	1.00		0.493	1.00		0.123
at place of birth	2	1	(0–11)	1.05	(0.76, 1.45)		1.36	(0.92, 2.02)	
in quintiles^h^	3	1	(0–10)	1.04	(0.75, 1.44)		1.55	(0.99, 2.42)	
	4	2	(0–8)	1.28	(0.93, 1.75)		1.87	(1.16, 3.02)	
	5	2	(0–12)	1.20	(0.87, 1.64)		1.85	(1.12, 3.04)	

^a^ adjusted for all other variables listed

^b^ IRR incidence rate ratio

^c^ 95% confidence interval

^d^ p-value from likelihood ratio test

^e^ number of children within a 250m radius around the residence of the child

^f^ any day-care attendance during first year of life

^g^ area based socio-economic position of the household

^h^ modelled annual average NO_2_ concentrations measured at place of birth (in μg/m^3^)

Having older siblings was associated with an increased likelihood of any respiratory symptoms of more than 50% (adjusted IRR 1 sibling: 1.60, 95%CI: 1.35–1.88; adjusted IRR 2+ siblings: 1.73, 95% CI: 1.40–2.13). Results were similar for lower respiratory tract infections (adjusted IRR 1 sibling: 1.62, 95%CI: 1.29–2.04; adjusted IRR 2+ siblings: 1.91, 95%CI: 1.44–2.52) (Table 2). Similarly, children who attended day-care during the first year of life more frequently had respiratory symptoms (adjusted IRR: 1.58, 95%CI: 1.32–1.89) and lower respiratory tract infections (adjusted IRR: 1.56, 95%CI: 1.23–1.98) than other children (Table 2). For breastfeeding, urbanity, SES and N0_2_ concentrations there was no evidence of associations with any outcome (Table 2).

### Interaction between number of siblings and neighbourhood child population density

There was no evidence of an interaction between siblings and neighbourhood child population density (dichotomised at median) regarding the frequency of respiratory symptoms (p LR tests >0.2, Table 3). Differences in IRRs between the 6 interaction levels were dominated by the effect of siblings: Having one or more older siblings was associated with an increased risk of respiratory symptoms, whereas differences associated with neighbourhood child density given the number of siblings was comparatively small (Table 3). Among children with 2 or more older siblings, higher child population density was even associated with lower risks compared to low child population density. For lower respiratory tract infections, we found weak evidence of an interaction (p LR test 0.055 adjusted model), however, high child population density tended to be associated again with lower incidence rates compared to low child population density in children with older siblings (Table 3).

**Table 3:** Interaction between child density and number of siblings as predictor of respiratory symptoms.

Interaction no. siblings & child	Number of infections		Crude models			Adjusted models^b^		
population density^a^	Median	Range	IRR^c^	95% CI^d^	p^e^	IRR^c^	95%CI ^d^	p^e^
**Any respiratory symptoms**	** **	** **	** **	** **	** **	** **	** **	** **
0 siblings x low density	3	(0–18)	1.00		0.449	1.00		0.233
0 siblings x high density	3	(0–22)	1.09	(0.87, 1.38)		1.00	(0.78, 1.28)	
1 siblings x low density	5	(0–23)	1.55	(1.21, 1.97)		1.59	(1.25, 2.02)	
1 siblings x high density	6	(0–21)	1.71	(1.35, 2.16)		1.57	(1.23, 2.01)	
2+ siblings x low density	6	(0–24)	1.73	(1.31, 2.29)		1.98	(1.50, 2.62)	
2+ siblings x high density	6	(0–15)	1.48	(1.08, 2.04)		1.41	(1.03, 1.94)	
**Lower respiratory tract infection**								
0 siblings x low density	1	(0–10)	1.00		0.128	1.00		0.055
0 siblings x high density	1	(0–9)	1.32	(0.96, 1.81)		1.13	(0.80, 1.59)	
1 siblings x low density	2	(0–12)	1.97	(1.42, 2.72)		2.12	(1.53, 2.92)	
1 siblings x high density	2	(0–10)	1.65	(1.20, 2.28)		1.44	(1.02, 2.03)	
2+ siblings x low density	2	(0–9)	1.97	(1.36, 2.86)		2.43	(1.67, 3.54)	
2+ siblings x high density	2	(0–10)	1.91	(1.26, 2.90)		1.69	(1.10, 2.60)	

^a^ median split of number of children within a 250m radius around the residence of the child

^b^ adjusted for day-care attendance, breastfeeding, urbanity, area based socio-economic position of the household, yearly average NO_2_ emissions measured at place of birth (in μg/m^3^)

^c^ IRR incidence rate ratio

^d^ 95% confidence interval

^e^ p-value from interaction siblings*density

### Sensitivity analyses

When we reduced the radius for calculating neighbourhood child population density from 250 m to 100 m, we found little evidence of association with any respiratory symptoms or with lower respiratory tract infections (S2 Table). When we increased the radius to 500m (S3 Table) or altered the age range to calculate neighbourhood population density to include only children below 10 years of age (S4 Table) or the total resident population, results were closely similar to those of our main analysis (S5 Table).

## Discussion

In a cohort study of healthy unselected infants with weekly assessments of respiratory symptoms throughout the first year of life, we found little evidence that living in a neighbourhood with a high childhood population density increased the frequency of respiratory symptoms or infections. Strong predictors for respiratory infections were the presence of siblings and attendance of day-care. There was little evidence for an interaction between the number of siblings and neighbourhood child density.

To our knowledge, no other study has investigated neighbourhood child density as a potential proxy for exposure to infections. An ecologic study from the UK found increased higher hospital admission rates for infections among children 0 to 14 years of age who lived in wards with high population density [9]. A number of studies have explored the transmission of infections in relation to patterns of social contact. A large survey of social contacts in eight European countries showed that schoolchildren and young adults have a high intensity of contacts and tend to mix with age peers. Contacts at home, school or leisure are likely to be physical. A mathematical model based on these data suggested that children aged 5–19 years suffer the highest incidence during the initial epidemic phase of an infection transmitted through social contacts [10]. Using the same data, Melegaro et al. found that intimate contacts (physical contacts, long-duration contacts, and frequent contacts) predicted the acquisition of serological markers for respiratory infections [21].

A number of studies have investigated the risk of childhood infections in relation to day-care attendance and family or household structure. Similar to our study, a Danish cohort study following children from birth to 1 year of age found a 50% increased risk of acute respiratory tract illnesses in children attending day-care. That study also found an increased risk of respiratory symptoms when having older siblings who were enrolled in day-care centres [22]. Another Danish cohort study found that children enrolled in day-care centres during the first 6 months of life had a 70% higher rate of hospitalisations for acute respiratory infections in the first year of life [6]. A large birth cohort study in the UK used latent class analysis to distinguish different patterns of infection in the first 6 months of life and found that having older children living in the same household was strongly associated with an increased risk of general infections [23]. Ponsonby et al. [8] compared several family size indicators in a cohort study and found that a higher number of persons per room increased the risk for lower respiratory tract infections in infants.

In our study, breastfeeding was not associated with respiratory infections in infancy. This contrasts with an earlier study of the same cohort [16] which found a protective effect of breastfeeding but only during the first six months after birth, and mainly in girls, whereas we investigated associations during the whole first year of life for both sexes. Furthermore, N0_2_ concentrations were only associated with lower respiratory infections when adjusted for other factors but not with any respiratory symptoms in crude or adjusted models. This is in line with other studies showing that high N0_2_ concentrations are an independent risk factor for chronic respiratory diseases and asthma [15,24,25].

The BILD cohort study, on which our analyses were based, prospectively assesses respiratory symptoms and infections on a weekly basis throughout the first year of life. Such intense follow-up throughout infancy is rare and provided us with an exceptional dataset to test our hypotheses. Parental recall is likely to be reliable over the short time interval of one week and the risk of recall bias was thus minimised. Precise geocodes were available for the entire Swiss population at census allowing us to calculate the precise number of children within a given radius from a study child. Furthermore, we had information on a number of competing exposures such as the number of older siblings, day-care attendance and breastfeeding as well as potential confounders such as degree of urbanity, neighbourhood SEP and NO_2_ concentrations, allowing us to appropriately adjust our analyses.

A limitation of our study is the rather small sample size which was not fully representative of the general population. Participating families were more likely to be well-educated and to belong to the middle or upper-class [13]. Hence there was lower variation in some of the relevant measures than would be expected in the general population: For example almost all mothers breastfed their children. Outcomes and most of the exposures (except those based on place of residence) were parent reported. However, interviews were conducted by trained study nurses. We had little information on non-respiratory infections and no information about parents’ social contacts. Since we could only enumerate the children living in a neighbourhood for the census years, we had to interpolate the exposure for children born in years between censuses. This might have caused some degree of misclassification of the exposure. In this study, neighbourhood child population density did not appear to be a valid proxy measure of exposure to infections: we found no evidence for an increase in the number of weeks with respiratory symptoms during the first year of life among infants from neighbourhoods with a high population density. A possible explanation is that in our study, families might be more likely to socialise with families from more distant neighbourhoods than with families from the same neighbourhood. We also found no effect modification by number of siblings. While our results do suggest that older siblings are a driver of respiratory infections in infancy, older siblings might primarily contract these infections from peers at school rather than in the neighbourhood. It is conceivable though that, in settings where social ties within neighbourhoods are stronger, child population density might be more strongly associated with early infections. Finally, child population density might only be a valid proxy of exposure to infections above a critical threshold level. Therefore, our findings may not apply to situations with higher (child) population density or with a higher degree of interaction within neighbourhoods.

## Conclusions

Our study suggests that in Switzerland, child population density in the neighbourhood is a poor proxy measure of exposure to respiratory infections in infants. While this measure has the advantage that it can be obtained from routine data, associations with respiratory symptoms in the first year of life were weak and inconsistent. These findings call into question the validity of population density for measuring early exposure to infections in studies investigating the hygiene hypothesis and other hypotheses regarding the role of early infections for normal immune development in high income countries.

## Supporting information

S1 Table Risk factors for severe respiratory symptoms. (PDF)

S2 Table Associations with child population density assessed within a 100 m radius. (PDF)

S3 Table Associations with child population density assessed within a 500 m radius. (PDF)

S4 Table Associations with neighbourhood density of children aged <10 years. (PDF)

S5 Table Associations with neighbourhood density of total population (all ages). (PDF)
