# Supplementary material for: Neighbourhood child population density as a proxy measure for exposure to respiratory infections in the first year of life: A validation study
Source: PLoS One. 2018 Sep 12;13(9):e0203743. doi: 10.1371/journal.pone.0203743 (PMC6135405; doi:10.1371/journal.pone.0203743)
Supplement: S1 Table — (PDF) [file pone.0203743.s001.pdf]

S1 Table: Severe respiratory symptoms and lower respiratory tract infections with fever: Child population density 250 m – children <16 years of age

		Number of infections		Crude models			Adjusted models[a]		
**Risk factor**		**Median**	**Range**	**IRR[b]**	**95%CI[c]**	**p[d]**	**IRR[b]**	**95%CI[c]**	**p[d]**
**Severe respiratory symptoms**									
Neighbourhood	1	0	(0 - 5)	1.00		0.006	1.00		0.003
child population	2	0	(0 - 11)	1.65	(1.06 , 2.55)		1.49	(0.89 , 2.47)	
density (250m)	3	0	(0 - 8)	0.79	(0.49 , 1.28)		0.68	(0.37 , 1.23)	
in quintiles[e]	4	0	(0 - 7)	0.82	(0.51 , 1.32)		0.63	(0.34 , 1.18)	
	5	0	(0 - 6)	0.91	(0.57 , 1.46)		0.75	(0.39 , 1.42)	
No. Siblings	0	0	(0 - 5)	1.00		<0.001	1.00		<0.001
	1	0	(0 - 11)	1.93	(1.39 , 2.67)		2.00	(1.44 , 2.79)	
	2	0	(0 - 5)	1.54	(1.02 , 2.33)		1.77	(1.17 , 2.68)	
Day-care attendance[f]	no	0	(0 - 8)	1.00		<0.001	1.00		<0.001
	yes	0.5	(0 - 11)	1.57	(1.13 , 2.19)		1.82	(1.29 , 2.57)	
Breastfeeding	never	0.5	(0 - 1)	0.61	(0.19 , 2.00)	0.486	0.47	(0.14 , 1.52)	0.372
	<= 6 mths	0	(0 - 11)	1.00			1.00		
	>6 mths	0	(0 - 8)	0.84	(0.61 , 1.17)		0.88	(0.64 , 1.21)	
Urbanity	rural	0	(0 - 11)	1.00		0.192	1.00		0.557
	urban	0	(0 - 8)	0.80	(0.56 , 1.12)		0.87	(0.54 , 1.39)	
SES in quintiles[g]	1	0	(0 - 4)	1.00		0.979	1.00		0.920
	2	0	(0 - 8)	1.13	(0.66 , 1.94)		1.04	(0.61 , 1.77)	
	3	0	(0 - 7)	0.98	(0.55 , 1.72)		0.90	(0.51 , 1.58)	
	4	0	(0 - 11)	1.07	(0.63 , 1.82)		0.95	(0.54 , 1.66)	
	5	0	(0 - 5)	1.01	(0.61 , 1.69)		0.84	(0.48 , 1.48)	
NO_2 level	1	0	(0 - 7)	1.00		0.599	1.00		0.622
at place of birth	2	0	(0 - 11)	1.29	(0.82 , 2.05)		1.30	(0.74 , 2.27)	
in quintiles[h]	3	0	(0 - 6)	1.02	(0.63 , 1.64)		1.46	(0.77 , 2.76)	
	4	0	(0 - 5)	1.10	(0.69 , 1.76)		1.68	(0.84 , 3.37)	
	5	0	(0 - 8)	0.89	(0.55 , 1.43)		1.32	(0.64 , 2.71)	
**Lower respiratory tract infection with fever**									
Neighbourhood	1	1	(0 - 6)	1.00		0.834	1.00		0.422
child population	2	1	(0 - 11)	1.09	(0.77 , 1.55)		1.05	(0.71 , 1.56)	
density (250m)	3	1	(0 - 5)	1.04	(0.73 , 1.48)		1.01	(0.65 , 1.56)	
in quintiles[e]	4	1	(0 - 4)	0.95	(0.66 , 1.36)		0.76	(0.48 , 1.22)	
	5	1	(0 - 5)	0.90	(0.63 , 1.29)		0.80	(0.48 , 1.31)	
No. Siblings	0	0	(0 - 5)	1.00		<0.001	1.00		<0.001
	1	1	(0 - 11)	1.62	(1.26 , 2.07)		1.67	(1.30 , 2.15)	
	2	1	(0 - 6)	1.43	(1.05 , 1.95)		1.58	(1.15 , 2.16)	
Day-care attendance[f]	no	0	(0 - 9)	1.00		<0.001	1.00		<0.001
	yes	1	(0 - 11)	1.60	(1.25 , 2.04)		1.72	(1.33 , 2.22)	
Breastfeeding	never	1	(0 - 3)	1.20	(0.55 , 2.60)	0.778	1.16	(0.54 , 2.47)	0.929
	<= 6 mths	1	(0 - 11)	1.00			1.00		
	>6 mths	1	(0 - 6)	0.95	(0.74 , 1.22)		1.02	(0.80 , 1.30)	
Urbanity	rural	0	(0 - 11)	1.00		0.141	1.00		0.012
	urban	1	(0 - 6)	0.82	(0.63 , 1.07)		0.62	(0.43 , 0.90)	
SES in quintiles[g]	1	1	(0 - 5)	1.00		0.024	1.00		0.079
	2	1	(0 - 9)	1.18	(0.79 , 1.78)		1.17	(0.78 , 1.75)	
	3	0	(0 - 4)	0.75	(0.48 , 1.17)		0.78	(0.50 , 1.22)	
	4	1	(0 - 11)	1.01	(0.68 , 1.52)		1.10	(0.72 , 1.69)	
	5	1	(0 - 6)	1.34	(0.92 , 1.95)		1.34	(0.88 , 2.05)	
NO_2 level	1	0	(0 - 6)	1.00		0.589	1.00		0.384
at place of birth	2	1	(0 - 11)	0.99	(0.69 , 1.41)		1.18	(0.77 , 1.79)	
in quintiles[h]	3	0	(0 - 4)	0.90	(0.63 , 1.30)		1.17	(0.71 , 1.92)	
	4	1	(0 - 5)	1.21	(0.86 , 1.71)		1.59	(0.95 , 2.67)	
	5	1	(0 - 4)	1.03	(0.72 , 1.46)		1.41	(0.82 , 2.44)	

[a] adjusted for all other variables listed
[b] IRR incidence rate ratio
[c] 95% confidence interval
[d] p-value from likelihood ratio test
[e] number of children within a 250m radius around the residence of the child
[f] any day-care attendance during first year of life
[g] area-based socio-economic position of the household
[h] modelled annual average NO_2 concentrations measured at place of birth (in $\mu g/m^3$)
