# Supplementary material for: Neighbourhood child population density as a proxy measure for exposure to respiratory infections in the first year of life: A validation study
Source: PLoS One. 2018 Sep 12;13(9):e0203743. doi: 10.1371/journal.pone.0203743 (PMC6135405; doi:10.1371/journal.pone.0203743)
Supplement: S2 Table — (PDF) [file pone.0203743.s002.pdf]

S2Table: Child population density 100 m – children <16 years of age

	Risk factor		Number of infections: Median	Range	Crude models: IRR [b]	95%CI [c]	p [d]	Adjusted models [a]: IRR [b]	95%CI [c]	p [d]
**Any respiratory symptoms**	Neighbourhood	1	4	(0 - 23)	1.00		0.956	1.00		0.952
	child population	2	4	(0 - 20)	0.99	(0.78 , 1.27)		1.03	(0.80 , 1.32)	
	density	3	4.5	(0 - 22)	1.00	(0.79 , 1.27)		0.93	(0.72 , 1.20)	
	in quintiles [e]	4	5	(0 - 24)	1.07	(0.85 , 1.36)		0.98	(0.76 , 1.26)	
		5	5	(0 - 21)	1.05	(0.83 , 1.34)		0.95	(0.72 , 1.25)	
**Lower respiratory tract infection**	Neighbourhood	1	1	(0 - 11)	1.00		0.955	1.00		0.886
	child population	2	1	(0 - 10)	0.92	(0.67 , 1.27)		0.97	(0.69 , 1.35)	
	density	3	1	(0 - 12)	0.95	(0.70 , 1.30)		0.87	(0.62 , 1.22)	
	in quintiles [e]	4	1.5	(0 - 11)	1.03	(0.76 , 1.39)		0.88	(0.62 , 1.23)	
		5	1	(0 - 10)	1.03	(0.76 , 1.41)		0.85	(0.59 , 1.23)	
**Severe respiratory symptoms**	Neighbourhood	1	0	(0 - 7)	1.00		0.329	1.00		0.261
	child population	2	0	(0 - 4)	0.82	(0.51 , 1.30)		0.86	(0.53 , 1.41)	
	density	3	0	(0 - 11)	1.03	(0.67 , 1.59)		0.83	(0.51 , 1.36)	
	in quintiles [e]	4	0	(0 - 8)	0.67	(0.42 , 1.06)		0.57	(0.34 , 0.96)	
		5	0	(0 - 5)	0.77	(0.49 , 1.21)		0.68	(0.39 , 1.17)	
**Lower respiratory tract infection**	Neighbourhood	1	1	(0 - 5)	1.00		0.846	1.00		0.959
**with fever**	child population	2	0	(0 - 6)	0.84	(0.59 , 1.21)		0.92	(0.63 , 1.33)	
	density	3	1	(0 - 11)	1.00	(0.72 , 1.41)		0.94	(0.65 , 1.36)	
	in quintiles [e]	4	1	(0 - 9)	0.93	(0.66 , 1.30)		0.86	(0.59 , 1.26)	
		5	1	(0 - 5)	1.01	(0.72 , 1.43)		0.92	(0.61 , 1.38)	

[a] adjusted for day-care attendance, number of siblings, breastfeeding, urbanity, area based socio-economic position of the household, yearly average NO_2 emissions measured at place of birth (in $\mu g/m^3$)
[b] IRR incidence rate ratio
[c] 95% confidence interval
[d] p-value from likelihood ratio test
[e] number of children within a 100 m radius around the residence of the child
