# Supplementary material for: Neighbourhood child population density as a proxy measure for exposure to respiratory infections in the first year of life: A validation study
Source: PLoS One. 2018 Sep 12;13(9):e0203743. doi: 10.1371/journal.pone.0203743 (PMC6135405; doi:10.1371/journal.pone.0203743)
Supplement: S3 Table — (PDF) [file pone.0203743.s003.pdf]

S3Table: Child population density 500 m – children <16 years of age

			Number of Infections		Crude models			Adjusted models [a]		
	Risk factor		Median	Range	IRR [b]	95%CI [c]	p [d]	IRR [b]	95%CI [c]	p [d]
**Any respiratory symptoms**	Neighbourhood	1	4	(0 - 24)	1.00		0.723	1.00		0.487
	child population	2	5	(0 - 22)	1.13	(0.89 , 1.45)		1.06	(0.81 , 1.40)	
	density	3	5	(0 - 21)	1.06	(0.83 , 1.35)		0.91	(0.66 , 1.26)	
	in quintiles [e]	4	6	(0 - 22)	1.18	(0.92 , 1.50)		1.12	(0.80 , 1.56)	
		5	4	(0 - 21)	1.07	(0.83 , 1.36)		0.97	(0.68 , 1.39)	
**Lower respiratory tract infection**	Neighbourhood	1	1	(0 - 11)	1.00		0.748	1.00		0.192
	child population	2	1	(0 - 11)	1.01	(0.73 , 1.38)		0.94	(0.66 , 1.34)	
	density	3	1	(0 - 8)	0.85	(0.62 , 1.18)		0.65	(0.42 , 1.00)	
	in quintiles [e]	4	2	(0 - 12)	1.06	(0.77 , 1.45)		0.83	(0.53 , 1.29)	
		5	2	(0 - 10)	1.00	(0.72 , 1.37)		0.69	(0.43 , 1.10)	
**Severe respiratory symptoms**	Neighbourhood	1	0	(0 - 11)	1.00		0.113	1.00		0.122
	child population	2	0	(0 - 8)	1.04	(0.66 , 1.62)		0.95	(0.57 , 1.57)	
	density	3	0	(0 - 5)	0.65	(0.40 , 1.04)		0.54	(0.29 , 1.02)	
	in quintiles [e]	4	0	(0 - 8)	1.04	(0.66 , 1.62)		0.86	(0.46 , 1.61)	
		5	0	(0 - 4)	0.69	(0.43 , 1.11)		0.57	(0.29 , 1.14)	
**Lower respiratory tract infection**	Neighbourhood	1	1	(0 - 11)	1.00		0.671	1.00		0.356
**with fever**	child population	2	1	(0 - 9)	0.81	(0.57 , 1.16)		0.78	(0.53 , 1.15)	
	density	3	1	(0 - 4)	0.79	(0.55 , 1.12)		0.60	(0.37 , 0.97)	
	in quintiles [e]	4	1	(0 - 4)	0.87	(0.62 , 1.24)		0.71	(0.43 , 1.15)	
		5	1	(0 - 5)	0.93	(0.66 , 1.31)		0.68	(0.41 , 1.13)	

[a] adjusted for day-care attendance, number of siblings, breastfeeding, urbanity, area based socio-economic position of the household, yearly average NO_2 emissions measured at place of birth (in $\mu g/m^3$)
[b] IRR incidence rate ratio
[c] 95% confidence interval
[d] p-value from likelihood ratio test
[e] number of children within a 500m radius around the residence of the child
