# Supplementary material for: Neighbourhood child population density as a proxy measure for exposure to respiratory infections in the first year of life: A validation study
Source: PLoS One. 2018 Sep 12;13(9):e0203743. doi: 10.1371/journal.pone.0203743 (PMC6135405; doi:10.1371/journal.pone.0203743)
Supplement: S4 Table — (PDF) [file pone.0203743.s004.pdf]

S4Table: Child population density 250 m – children <10 years of age

	Risk factor		Number of infections		Crude models			Adjusted models [a]		
			Median	Range	IRR [b]	95%CI [c]	p [d]	IRR [b]	95%CI [c]	p [d]
**Any respiratory symptoms**	Neighbourhood	1	4	(0 - 23)	1.00		0.641	1.00		0.655
	child population	2	4	(0 - 22)	1.16	(0.91 , 1.48)		1.14	(0.87 , 1.49)	
	density	3	5	(0 - 24)	1.14	(0.89 , 1.45)		1.20	(0.90 , 1.60)	
	in quintiles [e]	4	5	(0 - 20)	1.14	(0.89 , 1.45)		1.06	(0.78 , 1.45)	
		5	5	(0 - 22)	1.21	(0.94 , 1.54)		1.20	(0.86 , 1.67)	
**Lower respiratory tract infection**	Neighbourhood	1	1	(0 - 10)	1.00		0.894	1.00		0.736
	child population	2	1	(0 - 11)	1.14	(0.82 , 1.57)		1.09	(0.76 , 1.55)	
	density	3	1	(0 - 12)	1.11	(0.80 , 1.52)		1.08	(0.73 , 1.60)	
	in quintiles [e]	4	1	(0 - 7)	1.06	(0.77 , 1.46)		0.88	(0.58 , 1.33)	
		5	1	(0 - 10)	1.17	(0.85 , 1.61)		0.99	(0.64 , 1.54)	
**Severe respiratory symptoms**	Neighbourhood	1	0	(0 - 7)	1.00		0.476	1.00		0.214
	child population	2	0	(0 - 11)	1.17	(0.74 , 1.84)		0.98	(0.58 , 1.66)	
	density (100m)	3	0	(0 - 8)	1.06	(0.67 , 1.67)		0.98	(0.56 , 1.74)	
	in quintiles [e]	4	0	(0 - 7)	0.81	(0.50 , 1.30)		0.60	(0.32 , 1.10)	
		5	0	(0 - 5)	0.82	(0.51 , 1.33)		0.68	(0.35 , 1.32)	
**Lower respiratory tract infection**	Neighbourhood	1	1	(0 - 6)	1.00		0.994	1.00		0.532
**with fever**	child population	2	1	(0 - 11)	1.06	(0.75 , 1.52)		1.01	(0.68 , 1.48)	
	density (100m)	3	0	(0 - 5)	1.02	(0.72 , 1.45)		1.04	(0.67 , 1.59)	
	in quintiles [e]	4	1	(0 - 5)	0.98	(0.69 , 1.40)		0.77	(0.49 , 1.23)	
		5	1	(0 - 5)	1.02	(0.71 , 1.46)		0.84	(0.52 , 1.38)	

[a] adjusted for day-care attendance, number of siblings, breastfeeding, urbanity, area based socio-economic position of the household, yearly average NO_2 emissions measured at place of birth (in µg/m^3)
[b] IRR incidence rate ratio
[c] 95% confidence interval
[d] p-value from likelihood ratio test
[e] number of children within a 250m radius around the residence of the child
