# Supplementary material for: Neighbourhood child population density as a proxy measure for exposure to respiratory infections in the first year of life: A validation study
Source: PLoS One. 2018 Sep 12;13(9):e0203743. doi: 10.1371/journal.pone.0203743 (PMC6135405; doi:10.1371/journal.pone.0203743)
Supplement: S5 Table — (PDF) [file pone.0203743.s005.pdf]

S5Table: Population density 250 m – total population

	Risk factor		Number of infections: Median	Range	Crude models: IRR [b]	95%CI [c]	p [d]	Adjusted models [a]: IRR [b]	95%CI [c]	p [d]
**Any respiratory symptoms**	Neighbourhood	1	4	(0 - 23)	1.00		0.786	1.00		0.897
	child population	2	4	(0 - 24)	1.13	(0.89 , 1.45)		1.12	(0.86 , 1.47)	
	density	3	6	(0 - 21)	1.15	(0.90 , 1.46)		1.13	(0.83 , 1.52)	
	in quintiles [e]	4	5	(0 - 17)	1.15	(0.90 , 1.47)		1.07	(0.78 , 1.46)	
		5	4	(0 - 22)	1.12	(0.87 , 1.43)		1.05	(0.75 , 1.46)	
**Lower respiratory tract infection**	Neighbourhood	1	1	(0 - 11)	1.00		0.425	1.00		0.416
	child population	2	1	(0 - 12)	1.16	(0.84 , 1.59)		1.06	(0.74 , 1.51)	
	density	3	1	(0 - 9)	0.97	(0.70 , 1.34)		0.91	(0.61 , 1.36)	
	in quintiles [e]	4	2	(0 - 10)	1.22	(0.89 , 1.68)		1.00	(0.66 , 1.51)	
		5	1	(0 - 10)	0.95	(0.69 , 1.32)		0.76	(0.49 , 1.19)	
**Severe respiratory symptoms**	Neighbourhood	1	0	(0 - 7)	1.00		0.050	1.00		0.031
	child population	2	0	(0 - 11)	1.36	(0.87 , 2.10)		1.09	(0.65 , 1.83)	
	density	3	0	(0 - 4)	0.78	(0.49 , 1.24)		0.63	(0.35 , 1.13)	
	in quintiles [e]	4	0	(0 - 7)	0.84	(0.52 , 1.33)		0.62	(0.34 , 1.13)	
		5	0	(0 - 6)	0.73	(0.45 , 1.18)		0.51	(0.27 , 0.99)	
**Lower respiratory tract infection**	Neighbourhood	1	1	(0 - 6)	1.00		0.608	1.00		0.603
**with fever**	child population	2	1	(0 - 11)	1.18	(0.83 , 1.67)		1.14	(0.78 , 1.66)	
	density	3	0	(0 - 5)	0.95	(0.66 , 1.36)		0.99	(0.64 , 1.53)	
	in quintiles [e]	4	1	(0 - 5)	1.09	(0.77 , 1.55)		0.96	(0.61 , 1.50)	
		5	1	(0 - 5)	0.91	(0.63 , 1.32)		0.81	(0.50 , 1.33)	

[a] adjusted for day-care attendance, number of siblings, breastfeeding, urbanity, area based socio-economic position of the household, yearly average NO_2 emissions measured at place of birth (in µg/m^3)
[b] IRR incidence rate ratio
[c] 95% confidence interval
[d] p-value from likelihood ratio test
[e] number of people within a 250m radius around the residence of the child
